# Associations between red cell distribution width and in-hospital mortality in congestive heart failure patients with chronic obstructive pulmonary disease: a retrospective cohort study

**DOI:** 10.3389/fmed.2025.1448930

**Published:** 2025-05-20

**Authors:** Mixia Li, Kai Zhang, Yu Shi, Tianyi Cai, Zhao Xuan Gu, Yu Han, Fang Ming Gu, Tian Qi Zhang, Min Gao, Bo Li, Dan Cui, Kexiang Liu

**Affiliations:** ^1^Cardiovascular Surgery Department of the Second Hospital of Jilin University, Changchun, China; ^2^Department of Ophthalmology, Second Hospital of Jilin University, Changchun, China; ^3^Department of Ophthalmology, First Hospital of Jilin University, Changchun, China; ^4^Department of Cancer Center, The First Hospital of Jilin University, Changchun, China

**Keywords:** red cell distribution width, chronic obstructive pulmonary disease, congestive heart failure, in-hospital mortality, MIMIC

## Abstract

**Background:**

Recent studies have identified a co-occurrence of chronic obstructive pulmonary disease and congestive heart failure in ICU patients. Abnormal red cell distribution width (RDW) frequently manifests in critically ill patients, but its clinical significance remains a subject of debate. This study aims to investigate the relationship between RDW and in-hospital mortality in patients with concurrent congestive heart failure and chronic obstructive pulmonary disease.

**Methods:**

We conducted a retrospective cohort study using the Medical Information Mart for Intensive Care (MIMIC) IV version 2.0 database. RDW levels were assessed within 24 h of admission. The impact of RDW at ICU admission on in-hospital mortality was analyzed through multivariable logistic regression models, generalized additive models, and subgroup analysis.

**Results:**

We enrolled 6,309 patients with congestive heart failure and concomitant chronic obstructive pulmonary disease, with an in-hospital mortality rate of 12.4% (783/6,309). The fully adjusted model revealed a positive association between RDW and in-hospital mortality in congestive heart failure patients with concurrent chronic obstructive pulmonary disease, even after accounting for confounding factors (OR = 1.07, 95% CI: 1.03–1.12, *p* < 0.001). When comparing the highest quartile (Q4) to the lowest quartile (Q1), the odds ratio (OR) was 1.62, with a 95% confidence interval (CI) of 1.17–2.22, p = 0.003. We observed a linear relationship between RDW and in-hospital mortality, which remained consistent in subgroup analysis.

**Conclusions:**

Our data suggest that RDW is positively associated with in-hospital mortality in patients with both congestive heart failure and chronic obstructive pulmonary disease. At the same time, large prospective research and longer follow-up time are required to further validate the findings of this study.

## Introduction

Congestive heart failure (CHF) is the leading cause of morbidity and mortality worldwide, affecting about 4% of the adult population (1–3). Chronic obstructive pulmonary disease (COPD) is also a global health concern characterized by non-reversible airflow limitation (4). Its association with cardiovascular events underscores its significance as a cause of worldwide mortality (5, 6). COPD represents a heterogeneous collection of syndromes with overlapping features, complicating pathogenesis description (7). CHF and COPD frequently coexist due to shared risk factors (e.g., smoking, advanced age) and common pathophysiological mechanisms, such as the “cardiopulmonary continuum” and low-grade systemic inflammation (8–10). The strong link between heart failure and COPD has led to an increasing number of combined patient cases (11, 12). Despite this recognition, the precise factors connecting these conditions remain incompletely understood.

Red blood cell distribution width (RDW) measures the variation in size and volume of red blood cells (RBCs) (13). It serves as a simple, widely used, and cost-effective parameter for identifying various forms of anemia and reactive bone marrow states (14). Traditionally employed in laboratory hematology to differentially diagnose anemias, RDW is now backed by substantial evidence linking anisocytosis to various human disorders. Recent studies have shown RDW's association with adverse outcomes in critical illness and heart diseases. In chronic ambulatory heart failure patients, RDW emerges as one of the strongest predictors of mortality, even after adjusting for commonly used disease severity indices (15, 16). RDW has also been validated as a marker for adverse outcomes in acute decompensated heart failure and coronary artery bypass grafting surgery patients (17–19). Notably, Sincer et al. reported that high RDW was an independent predictor of right ventricular failure (odds ratio: 2.098; *P* = 0.017), demonstrating a sensitivity of 70% and specificity of 93.1% (20). Four studies established significant and independent associations between RDW and mortality in COPD patients (21–24). Seyhan et al. found an independent association between RDW and five-year mortality (hazard ratio, HR = 1.12; 95% CI 1.01 to 1.24; *p* = 0.01) (21). Tertemiz et al. similarly found an independent association with nine-year mortality (HR = 1.222, 95% CI 1.153 to 1.295, *p* < 0.01) (22). Lan et al. retrospectively analyzed an association between the upper RDW tertile and 28-day mortality (OR = 1.70, 95% CI 1.29 to 2.22, *p* = 0.0001) (23). Finally, Qiu et al. demonstrated an independent association between RDW and ten-year mortality (HR = 1.12, 95% CI 1.00 to 1.25, *p* = 0.046) (24).

Nevertheless, despite a strong link between heart failure and COPD, leading to an increasing number of combined patient cases (11, 12). However, no study has indicated the relationship between RDW and clinical outcomes in patients with COPD and CHF. Therefore, in our study, we aimed to explore the association between RDW and mortality in CHF and COPD based on a large-scale public database.

## Method

### Data source

This study is a retrospective observational analysis using data extracted from the Medical Information Mart for Intensive Care IV (MIMIC-IV), an international online database (25). The MIMIC-IV database is publicly available through the Massachusetts Institute of Technology's Computational Physiology Laboratory (MIT, Cambridge, Massachusetts, USA). It contains clinical information from patients admitted to the ICU at Beth Israel Deaconess Medical Center (BIDMC, Boston, MA, USA) (26). Data extraction from MIMIC-IV was conducted following completion of the National Institutes of Health (NIH) web-based training course and the Protecting Human Research Participants examination (author certification number: 11639604). Ethical approval for the MIMIC-IV database was obtained from the institutional review boards of MIT and BIDMC (27). This study received approval from the institutional review boards of MIT and BIDMC and was granted a waiver of informed consent (28). The reporting of this study adheres to the Strengthening the Reporting of Observational Studies in Epidemiology (STROBE) statement.

### Study population

**Figure 1:**
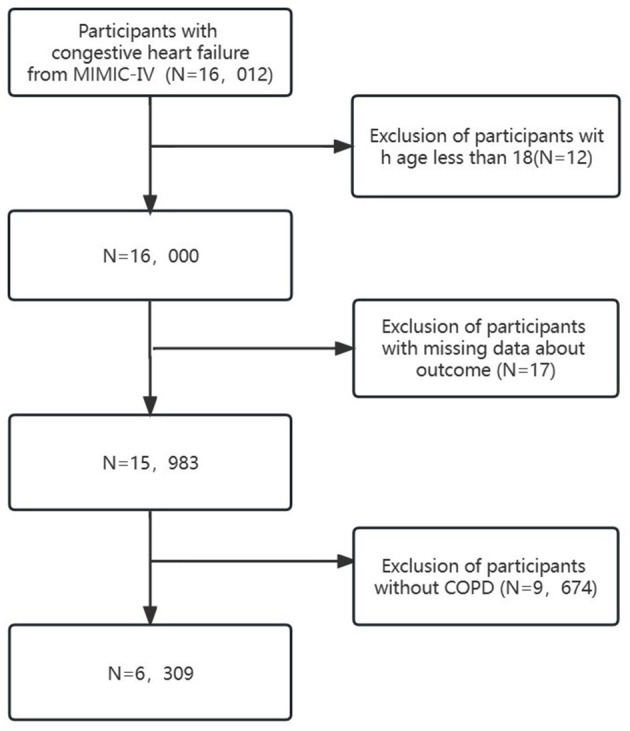
Flowchart of patient selection.

Clinical data from the MIMIC-IV database were extracted using structured query language (SQL) programming in Navicat Premium (version 15). Patients diagnosed with “CHF” based on International Classification of Diseases editions (ICD-9 and ICD-10) diagnostic codes were included (see Supplementary Table S1). Exclusion criteria comprised (1) patients under 18 years old, (2) patients with missing outcome data, and (3) patients without COPD. Ultimately, 6,309 patients were included (Figure 1).

### Variates

The initial red blood cell distribution width was treated as a continuous variable. Based on the database and previous research, In the present research, we reported the RDW-CV and represented it with RDW. Patients were categorized into three groups based on quartiles of red blood cell distribution width on the first day of ICU admission: Q1 (≤14.2), Q2 (14.2–15.4), Q3 (15.4–17.1), and Q4 (>17.1). The primary outcome assessed was in-hospital mortality, determined by the patient's survival status at discharge.

Based on prior literature and clinical insights, the following covariates were identified: (1) Demographic variables, including sex, age, and race; (2) Comorbidities such as chronic obstructive acute myocardial infarction (AMI), diabetes, hepatic failure (Hep F), Melanosis coli (MC), and diabetes; (3) Medical procedures, including ventilation and intubation; (4) Medication usage, including norepinephrine, dopamine, epinephrine, phenylephrine, and vasopressin; (5) Basic vital signs, such as temperature, respiratory rate, heart rate, systolic blood pressure (SBP); (6) Blood biochemical indicators, including anion gap (AG), blood urea nitrogen (BUN), chloride, creatinine, hemoglobin (Hb), mean corpuscular hemoglobin (MCH), mean corpuscular hemoglobin concentration (MCHC), mean corpuscular volume (MCV), platelet count, potassium, sodium, red blood cell (RBC) count, and white blood cell (WBC) count; and (7) Acute Physiology Score III (APSIII) and Sequential Organ Failure Assessment Score (SOFA).

### Statistical analyses

In this study, we categorized participants into four groups based on the interquartile range of RDW for descriptive analysis. Continuous variables were summarized as mean and standard deviation (SD) for normally distributed data, or median and interquartile range for skewed distributions. We employed the chi-square test for categorical variables, ANOVA for normally distributed continuous variables, and the Kruskal-Wallis test for non-normally distributed continuous variables.

To assess the association between RDW and in-hospital mortality in patients with Congestive Heart Failure with Concomitant COPD, we conducted multivariate regression analyses using five models. Model 1 was unadjusted, while Model 2 adjusted for demographic variables (sex, age, race). Model 3 included adjustments for demographic variables and comorbidities (diabetes, AMI, MC, HepF). Model 4 considered demographic variables, comorbidities, medical procedures (ventilation and intubation), medication status (norepinephrine, dopamine, epinephrine, phenylephrine, vasopressin), vital signs (temperature, respiratory rate, heart rate, SBP), and blood biochemical indicators (AG, BUN, chloride, creatinine, Hb, MCH, MCHC, MCV, platelet, potassium, sodium, RBC, WBC). Lastly, Model 5 adjusted for demographic variables, comorbidities, medical procedures, medication status, vital signs, blood biochemical indicators, APS III, and SOFA.

To assess significant associations, we employed the generalized additive model and fitted smooth curves using the penalty spline method to examine the dose-response relationship. Subgroup analysis, conducted through hierarchical logistic regression, evaluated potential modifiers of the RDW-in-hospital mortality association, including sex, age (<60 vs. ≥60 years), race (white vs. black vs. other), norepinephrine (Yes vs. No), dopamine (Yes vs. No), epinephrine (Yes vs. No), phenylephrine (Yes vs. No), vasopressin (Yes vs. No), AMI (Yes vs. No), diabetes (Yes vs. No), and hepatic failure (Yes vs. No).

All statistical analyses were performed using R 4.2.2 (http://www.R-project.org, The R Foundation) and Free Statistics software version 1.7.1. Two-tailed tests were conducted with a significance level of P < 0.05, indicating statistical significance. This cross-sectional study adhered to the Strengthening the Reporting of Observational Studies in Epidemiology (STROBE) statement for reporting.

## Results

### Baseline characteristics of selected participants

This study encompassed 6,309 patients, with an in-hospital mortality rate of 12.4%. Tables 1, 2 presents the baseline characteristics of these patients. The mean age of the cohort was 73.0 ± 12.5 years, with 48.5% (3,063) being male. Based on their baseline RDW levels, participants were stratified into four groups: Q1 (≤14.2), Q2 (14.2–15.4), Q3 (15.4–17.1), and Q4 (>17.1).

**Table 1:** Characteristics of the study population (N = 6,309).

**Variables**	**Total (*n* = 6,309)**	**Q1 (*n* = 1,492)^b^**	**Q2 (*n* = 1,588)^b^**	**Q3 (*n* = 1,631)^b^**	**Q4 (*n* = 1,598)^b^**	***P* value^a^**
Age, mean ± SD	73.0 ± 12.5	72.7 ± 12.7	73.8 ± 12.3	73.1 ± 12.4	72.3 ± 12.4	0.007
**Gender**, ***n*** **(%)**						0.007
Male	3,063 (48.5)	768 (51.5)	759 (47.8)	809 (49.6)	727 (45.5)	
Female	3,246 (51.5)	724 (48.5)	829 (52.2)	822 (50.4)	871 (54.5)	
**Race**, ***n*** **(%)**						<0.001
White	4,339 (68.8)	1,029 (69)	1,128 (71)	1,128 (69.2)	1,054 (66)	
Black	901 (14.3)	155 (10.4)	203 (12.8)	245 (15)	298 (18.6)	
Other	1,069 (16.9)	308 (20.6)	257 (16.2)	258 (15.8)	246 (15.4)	
**Medication situation**						
**Norepinephrine**, ***n*** **(%)**						<0.001
No	4,883 (77.4)	1,200 (80.4)	1,276 (80.4)	1,237 (75.8)	1,170 (73.2)	
Yes	1,426 (22.6)	292 (19.6)	312 (19.6)	394 (24.2)	428 (26.8)	
**Dopamine**, ***n*** **(%)**						0.303
No	6,126 (97.1)	1,447 (97)	1,552 (97.7)	1,583 (97.1)	1,544 (96.6)	
Yes	183 (2.9)	45 (3)	36 (2.3)	48 (2.9)	54 (3.4)	
**Epinephrine, n (%)**						0.011
No	6,086 (96.5)	1,419 (95.1)	1,540 (97)	1,576 (96.6)	1,551 (97.1)	
Yes	223 (3.5)	73 (4.9)	48 (3)	55 (3.4)	47 (2.9)	
**Phenylephrine**, ***n*** **(%)**						0.012
No	5,859 (92.9)	1,403 (94)	1,489 (93.8)	1,507 (92.4)	1,460 (91.4)	
Yes	450 (7.1)	89 (6)	99 (6.2)	124 (7.6)	138 (8.6)	
**Vasopressin**, ***n*** **(%)**						0.005
No	5,901 (93.5)	1,410 (94.5)	1,501 (94.5)	1,523 (93.4)	1,467 (91.8)	
Yes	408 (6.5)	82 (5.5)	87 (5.5)	108 (6.6)	131 (8.2)	
**Medical procedures**						
**Vent**, ***n*** **(%)**						<0.001
No	678 (10.7)	161 (10.8)	145 (9.1)	159 (9.7)	213 (13.3)	
Yes	5,631 (89.3)	1,331 (89.2)	1,443 (90.9)	1,472 (90.3)	1,385 (86.7)	
**Intubated**, ***n*** **(%)**						<0.001
No	4,412 (69.9)	1,015 (68)	1,095 (69)	1,121 (68.7)	1,181 (73.9)	
Yes	1,897 (30.1)	477 (32)	493 (31)	510 (31.3)	417 (26.1)	

%, weighted proportion; Hstatus, hospital status; CHF, congestive heart failure; COPD, chronic obstructive pulmonary disease; Hep F, hepatic failure; AMI, acute myocardial infarction; APSIII, Acute Physiology III; SOFA, Sequential Organ Failure Assessment; SBP, systolic blood pressure; AG, anion gap; BUN, blood urea nitrogen; MCH, mean corpuscular hemoglobin; MCHC, mean corpuscular hemoglobin concentration; MCV, mean corpuscular volume; RBC, red blood cell; RDW, red blood cell distribution width; WBC, white blood cell count.

Q1 (≤14.2); Q2 (14.2–15.4); Q3 (15.4–17.1); Q4 (>17.1).

^a^P values of multiple comparisons were corrected by the False Discovery Rate method.

^b^Q1–Q4: according to RDW.

**Table 2:** Other characteristics of the study population (*N* = 6,309).

**Variables**	**Total (*n* = 6,309)**	**Q1 (*n* = 1,492)^b^**	**Q2 (*n* = 1,588)^b^**	**Q3 (*n* = 1,631)^b^**	**Q4 (*n* = 1,598)^b^**	***P* value^a^**
**complicating disease**						
**Diabetes**, ***n*** **(%)**						<0.001
No	3,567 (56.5)	935 (62.7)	903 (56.9)	901 (55.2)	828 (51.8)	
Yes	2,742 (43.5)	557 (37.3)	685 (43.1)	730 (44.8)	770 (48.2)	
**AMI**, ***n*** **(%)**						0.004
No	4,509 (71.5)	1,022 (68.5)	1,125 (70.8)	1,174 (72)	1,188 (74.3)	
Yes	1,800 (28.5)	470 (31.5)	463 (29.2)	457 (28)	410 (25.7)	
**MC**, ***n*** **(%)**						<0.001
No	5,661 (89.7)	1,381 (92.6)	1,447 (91.1)	1,461 (89.6)	1,372 (85.9)	
Yes	648 (10.3)	111 (7.4)	141 (8.9)	170 (10.4)	226 (14.1)	
**Hep F**, ***n*** **(%)**						<0.001
No	6,152 (97.5)	1,485 (99.5)	1,567 (98.7)	1,596 (97.9)	1,504 (94.1)	
Yes	157 (2.5)	7 (0.5)	21 (1.3)	35 (2.1)	94 (5.9)	
**Vital signs**						
Temperature, mean ± SD	36.7 ± 0.5	36.8 ± 0.5	36.8 ± 0.4	36.7 ± 0.5	36.7 ± 0.5	<0.001
Respiratory rate, mean ± SD	20.1 ± 3.7	19.9 ± 3.6	20.2 ± 3.8	20.1 ± 3.7	20.2 ± 3.8	0.136
Heart rate, mean ± SD	84.7 ± 15.6	84.6 ± 14.8	84.4 ± 15.5	84.0 ± 15.9	85.7 ± 16.0	0.02
SBP, mean ± SD	116.5 ± 16.4	118.5 ± 16.4	117.8 ± 16.6	116.1 ± 16.1	113.9 ± 16.2	<0.001
**Blood biochemical indicators**						
AG, mean ± SD	14.9 ± 4.1	14.3 ± 3.8	14.6 ± 3.9	14.9 ± 4.0	15.6 ± 4.4	<0.001
BUN, mean ± SD	35.4 ± 24.4	27.6 ± 18.3	33.4 ± 23.4	38.0 ± 25.3	42.0 ± 27.2	<0.001
Creatinine, mean ± SD	1.8 ± 1.6	1.4 ± 1.0	1.7 ± 1.5	1.9 ± 1.8	2.1 ± 1.7	<0.001
Hb, mean ± SD	10.2 ± 2.1	11.2 ± 2.1	10.5 ± 2.0	10.0 ± 1.9	9.3 ± 2.0	<0.001
MCH, mean ± SD	29.3 ± 2.9	30.6 ± 2.0	29.7 ± 2.2	29.0 ± 2.7	28.0 ± 3.7	<0.001
MCHC, Mean ± SD	32.1 ± 1.8	32.9 ± 1.4	32.4 ± 1.6	31.9 ± 1.6	31.1 ± 1.9	<0.001
MCV, Mean ± SD	91.4 ± 7.6	93.3 ± 5.9	91.7 ± 6.2	91.1 ± 7.4	89.7 ± 9.8	<0.001
Platelet, mean ± SD	215.9 ± 100.1	212.3 ± 88.7	216.5 ± 95.8	218.9 ± 100.7	215.7 ± 112.9	0.32
Potassium, mean ± SD	4.3 ± 0.8	4.3 ± 0.7	4.3 ± 0.8	4.4 ± 0.8	4.4 ± 0.8	0.009
Sodium, mean ± SD	138.2 ± 5.1	138.1 ± 4.8	138.3 ± 5.1	138.4 ± 5.1	137.9 ± 5.4	0.02
RBC, mean ± SD	3.5 ± 0.7	3.7 ± 0.7	3.5 ± 0.7	3.5 ± 0.7	3.4 ± 0.9	<0.001
Calcium, mean ± SD	8.5 ± 0.8	8.5 ± 0.8	8.5 ± 0.8	8.5 ± 0.8	8.5 ± 0.8	0.201
Chloride, mean ± SD	101.4 ± 6.9	102.2 ± 6.6	101.8 ± 6.7	101.3 ± 6.8	100.4 ± 7.1	<0.001
WBC, mean ± SD	11.8 ± 8.6	12.0 ± 7.2	11.6 ± 9.6	11.5 ± 7.0	12.0 ± 10.1	0.301
SOFA, mean ± SD	3.3 ± 2.8	2.9 ± 2.6	3.2 ± 2.7	3.4 ± 2.9	3.7 ± 3.1	<0.001
APSIII, mean ± SD	51.9 ± 21.3	46.2 ± 19.8	51.2 ± 21.1	53.5 ± 21.2	56.2 ± 21.7	<0.001
**Hstatus**, ***n*** **(%)**						<0.001
Survival	5,526 (87.6)	1,374 (92.1)	1,428 (89.9)	1,409 (86.4)	1,315 (82.3)	
Death	783 (12.4)	118 (7.9)	160 (10.1)	222 (13.6)	283 (17.7)	

%, weighted proportion; Hstatus, hospital status; CHF, congestive heart failure; COPD, chronic obstructive pulmonary disease; Hep F, hepatic failure; AMI, acute myocardial infarction; APSIII, Acute Physiology III; SOFA, Sequential Organ Failure Assessment; SBP, systolic blood pressure; AG, anion gap; BUN, blood urea nitrogen; MCH, mean corpuscular hemoglobin; MCHC, mean corpuscular hemoglobin concentration; MCV, mean corpuscular volume; RBC, red blood cell; RDW, red blood cell distribution width; WBC, white blood cell count.

Q1 (≤14.2); Q2 (14.2–15.4); Q3 (15.4–17.1); Q4 (>17.1).

^a^P values of multiple comparisons were corrected by the False Discovery Rate method.

^b^Q1-Q4: according to RDW.

Furthermore, individuals with higher RDW levels, as opposed to those with lower levels, exhibited an elevated proportion of Black participants, larger diameters, lower AMI rates, higher MC and HepF rates, lower body temperature, decreased SBP, higher AG, BUN, and creatinine levels, lower hemoglobin (Hb), MCH, MCHC, MCV, higher potassium levels, decreased RBC counts, and lower blood chlorine levels. Additionally, higher RDW levels were associated with an increased SOFA score, APS score, and mortality rate when compared to individuals with lower RDW levels.

### Association between RDW and in-hospital mortality in COPD patients with congestive heart failure

Table 3 displays the outcomes of a multivariable logistic regression analysis that investigates the association between RDW and in-hospital mortality in patients concurrently diagnosed with both heart failure and COPD. The analysis presents odds ratios (ORs) with corresponding 95% confidence intervals (CIs) across various RDW levels, elucidating the in-hospital mortality risk within this patient cohort.

**Table 3:** Multivariable logistic regression to assess the association of RDW with in-hospital mortality rate.

	**Model 1**		**Model 2**		**Model 3**		**Model 4**		**Model 5**	
**RDW**	**OR_95CI**	**P value**	**OR_95CI**	**P value**	**OR_95CI**	**P value**	**OR_95CI**	**P value**	**OR_95CI**	***P*** **value**
Continuous variable	1.13 (1.1–1.17)	<0.001	1.15 (1.12–1.18)	<0.001	1.14 (1.11–1.18)	<0.001	1.08 (1.04–1.13)	<0.001	1.07 (1.03–1.12)	0.001
**Categorical variable**										
Q1 (≤14.2)	1 (Ref)		1 (Ref)		1 (Ref)		1 (Ref)		1 (Ref)	
Q2 (14.2-15.4)	1.3 (1.02–1.67)	0.036	1.3 (1.01–1.67)	0.04	1.3 (1.01–1.67)	0.043	1.19 (0.89–1.59)	0.249	1.03 (0.76–1.4)	0.859
Q3 (15.4-17.1)	1.83 (1.45–2.32)	<0.001	1.88 (1.49–2.39)	<0.001	1.86 (1.47–2.37)	<0.001	1.5 (1.13–2)	0.005	1.34 (0.99–1.81)	0.058
Q4 (>17.1)	2.51 (2–3.15)	<0.001	2.67 (2.12–3.37)	<0.001	2.55 (2.02–3.23)	<0.001	1.78 (1.31–2.41)	<0.001	1.62 (1.17–2.22)	0.003
*P* for tread		<0.001		<0.001		<0.001		<0.001		0.001

The unit of RDW is %. %, weighted proportion; CHF, congestive heart failure; COPD, chronic obstructive pulmonary disease; HepF, hepatic failure; AMI, acute myocardial infarction; APSIII, Acute Physiology III; SOFA, Sequential Organ Failure Assessment; SBP, systolic blood pressure; AG, anion gap; BUN, blood urea nitrogen; MCH, mean corpuscular hemoglobin; MCHC, mean corpuscular hemoglobin concentration; MCV, mean corpuscular volume; RBC, red blood cell; RDW, red blood cell distribution width; WBC, white blood cell count; CI, confidence interval; OR, odds ratios; Ref, reference.

Model 1: No adjustment.

Model 2: Adjusted for demographic variables (sex, age, race).

Model 3: Adjusted for demographic variables, comorbidities (diabetes, AMI, MC, HepF).

Model 4: Adjusted for demographic variables, comorbidities, Medical Procedures (Vent, Intubated), Medication situation (Norepinephrine Dopamine Epinephrine Phenylephrine Vasopressin), Basic vital signs (Temperature Respiratory Rate Heart Rate SBP), Blood biochemical indicators (AG BUN Chloride Creatinine, Hb MCH MCHC MCV Platelet Potassium Sodium RBC WBC).

Model 5: Adjusted for demographic variables, comorbidities, Medical Procedures, Medication situation, Basic vital signs, Blood biochemical indicators, APSIII, SOFA.

In the fully adjusted model treating RDW as a continuous variable, each unit increase in RDW correlates with a 7% increase in the risk of in-hospital mortality (OR = 1.07; 95% CI = 1.03–1.12; *p* < 0.001). When RDW levels are categorized into four groups, a significant positive association between RDW and in-hospital mortality persists after accounting for potential confounders. Compared to individuals in Q1, the adjusted OR values for RDW and in-hospital mortality in Q2, Q3, and Q4 are 1.03 (95% CI: 0.76–1.4, *p* = 0.859), 1.34 (95% CI: 0.99–1.81, *p* = 0.058), and 1.62 (95% CI: 1.17–2.22, *p* = 0.003), respectively (refer to Table 3). This trend demonstrates statistical significance (P for trend = 0.001).

#### Dose–response relationships

**Figure 2:**
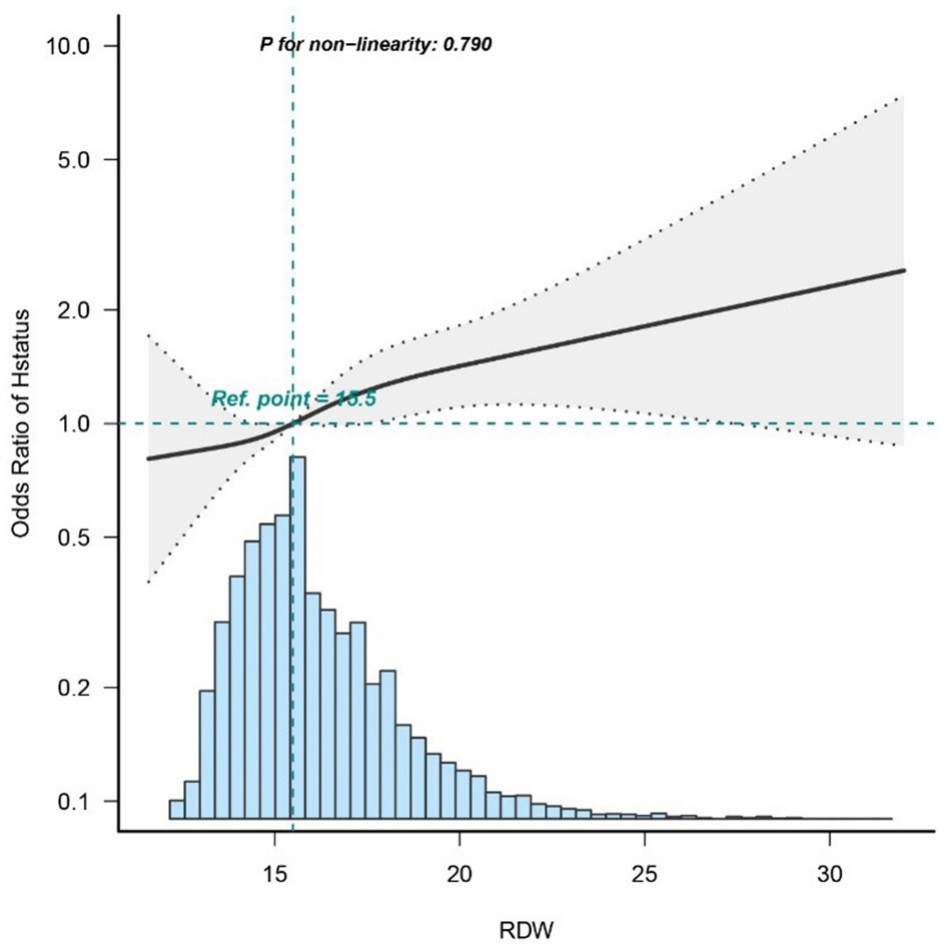
Dose–response relationships between RDW with intrahospital mortality rate odds ratio.

To investigate the association between RDW and in-hospital mortality, a logistic regression model with a cubic spline function was utilized. Figure 2 depicts variable distributions (blue histograms), the smoothing curve (solid black line) representing the relationship between the variables, and the 95% confidence interval (gray shaded area). Following adjustment for confounding factors, a statistically significant linear correlation is observed between RDW and in-hospital mortality (*p* = 0.79).

#### Subgroup analysis

**Figure 3:**
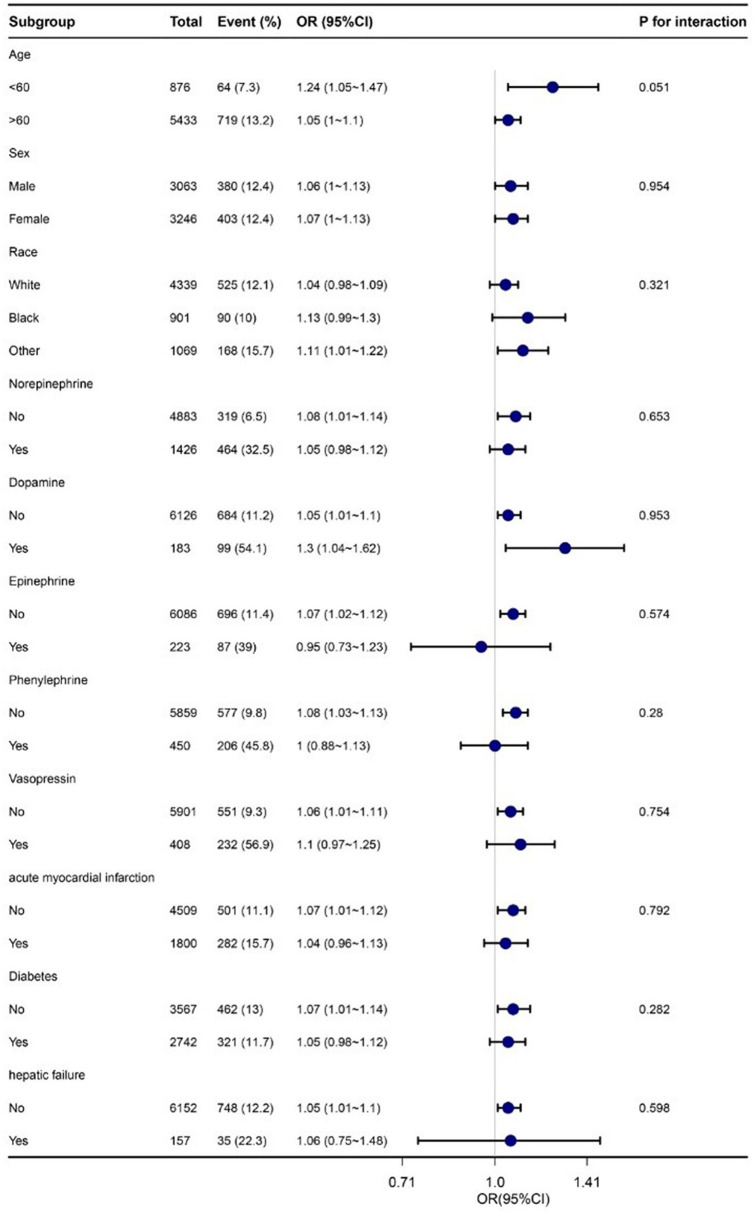
Stratified analyses of the association between RDW with in-hospital mortality rate.

Subgroup analyses were conducted to evaluate potential modifications in the relationship between RDW and in-hospital mortality within various subgroups. No significant interactions were found in any of the subgroups, whether stratified by age, sex, race, norepinephrine, dopamine, epinephrine, phenylephrine, vasopressin, acute myocardial infarction, diabetes, or hepatic failure (refer to Figure 3). It should be noted that, considering multiple testing, the *p*-value for the interaction of norepinephrine and phenylephrine may not reach statistical significance if it is <0.05.

## Discussion

We conducted a retrospective cohort study using the MIMIC-IV database. This study is the first to provide evidence of a linear correlation between RDW and In-Hospital Mortality among COPD patients with Congestive Heart Failure in the United States. Our pioneering findings reveal a significant positive association between RDW levels and In-Hospital Mortality, with a 7% increase in mortality for every 1% increase in RDW. Even after adjusting for confounding factors, RDW remains significantly associated with In-Hospital Mortality. These results underscore the importance of monitoring RDW levels in ICU-admitted COPD patients with Congestive Heart Failure, offering valuable insights to clinicians.

The Red Cell Distribution Width (RDW) is a metric reflecting the variability in erythrocyte size, and it serves as a readily accessible hematologic inflammatory marker. An accumulating body of evidence underscores its significant prognostic value across various medical conditions. Specifically, prior research has identified elevated RDW levels in patients with diabetes, chronic kidney disease, anemia, inflammatory-related disorders such as infection and cancer, and heart failure (29–33). Moreover, investigations have demonstrated the predictive power of RDW in both chronic and acute left heart failure (HF) (15). For instance, Hampole et al. reported an independent association between RDW and mortality in 162 patients with pulmonary hypertension (PH), suggesting its superior prognostic utility compared to N-terminal pro-natriuretic peptide (34). Similarly, RDW emerged as a robust independent prognostic indicator in PH, surpassing natriuretic peptides (35). Additionally, Van Kimmenade et al. provided insights into the potential augmentation of prognostic information by RDW when combined with N-terminal pro-natriuretic peptide in acute heart failure (36). In large cohorts of congestive heart failure patients, Felker et al. (15) revealed the independence of RDW as a predictor for both morbidity and mortality. Furthermore, in chronic obstructive pulmonary disease (COPD) patients, Seyhan et al. (21) identified an independent association between RDW and five-year mortality, while Tertemiz et al. (22) extended this association to nine-year mortality. Lan et al. (23) conducted a retrospective analysis, observing a significant link between elevated RDW levels and 28-day mortality in COPD patients. Lastly, Qiu et al. (24) demonstrated an independent association between RDW and 10-year mortality in COPD patients. Notably, this study represents the first documentation of an association between RDW and In-Hospital Mortality in COPD Patients with Congestive Heart Failure in the Intensive Care Unit (ICU). Utilizing multivariate regression analysis, we confirmed the association between RDW and In-Hospital Mortality. Importantly, this association persisted when RDW was transformed into a categorical variable, underscoring the clinical significance of RDW as a common indicator that warrants close attention from ICU physicians.

The mechanistic link between RDW and poor prognosis in chronic diseases such as congestive heart failure (CHF) and chronic obstructive pulmonary disease (COPD) remains incompletely elucidated. Oxidative stress and chronic inflammation are posited as pivotal factors in this association. Erythrocytes serve the dual functions of oxygen delivery to tissues and participation in cardiovascular regulation through the release of extracellular nucleotides and other mediators (37). Thus, it is conceivable that alterations in erythrocyte function could directly impact cardiac and pulmonary function. Evidence suggests that chronic inflammatory conditions may induce inefficient erythropoiesis, resulting in the entry of immature red blood cells (RBCs) into circulation and subsequently elevating RDW (38). Inflammatory markers such as tumor necrosis factor-α and interleukin-6 can disrupt erythropoiesis through mechanisms including myelosuppression of erythroid precursors, reduced iron bioavailability for hemoglobin synthesis, promotion of apoptosis, increased erythropoietin resistance in precursor cell lines, and diminished erythropoietin production from the kidney (39, 40). Erythrolysis, on the other hand, is associated with an upsurge in the production of free radicals, which are known to exert detrimental effects on cardiac and pulmonary function (41). Elevated oxidative stress represents another potential pathway linking RDW with mortality, as it diminishes RBC survival and results in anisocytosis due to an increase in circulating premature erythrocytes (42, 43). A comprehensive understanding of the biological mechanisms underpinning the association between RDW and multiple health outcomes may facilitate the identification of potential therapeutic targets (44). This cost-effective and readily available parameter holds promise in providing valuable insights into individuals' health status, the presence of both subclinical and clinical diseases, and prognostication in diverse acute and chronic conditions. Irrespective of the underlying etiology, individuals with decreased RDW values should receive heightened monitoring and more intensive management to enhance clinical outcomes.

Dietary and lifestyle modifications can help reduce red blood cell distribution width (RDW), a marker of cardiovascular disease risk (45). Adopting a Mediterranean diet, which is high in monounsaturated fatty acids (MUFAs) and polyunsaturated fatty acids (PUFAs), can help reduce inflammation and improve cardiovascular health (46). Engaging in regular physical activity and weight management can also contribute to lowering RDW by improving overall health and reducing cardiovascular disease risk factors (47). Consuming a balanced diet rich in fruits, vegetables, whole grains, and legumes, while limiting red and processed meats and alcohol, can help reduce the risk of cancer and other chronic diseases (48). By implementing these dietary and lifestyle modifications, individuals can potentially lower their RDW and improve Mortality among COPD patients with Congestive Heart Failure.

The methodology employed in this study offers distinct advantages. Firstly, previous investigations into risk factors and prognostic factors for congestive heart failure (CHF) and chronic obstructive pulmonary disease (COPD) have often been limited by small sample sizes. To the best of our knowledge, this study represents the initial endeavor to analyze CHF and COPD patients within the MIMIC-IV database. Secondly, we employed smoothing function analysis to address potential data irregularities, thereby enhancing our comprehension of the relationship between RDW levels and in-hospital mortality. To mitigate the impact of confounding factors inherent in observational studies, we conducted logistic regression analysis using multiple models, supplemented by subgroup analyses for appropriate stratification.

Nevertheless, it is imperative to acknowledge specific limitations in our study. Primarily, the retrospective research design utilized may compromise the validity of our findings, necessitating validation through future prospective case-control studies. Furthermore, the incompleteness of publicly available databases impeded the collection of variables that could influence the model and its outcomes. In forthcoming research, we intend to utilize our proprietary database to address this limitation and explore related areas more comprehensively. Lastly, the exclusive inclusion of American participants restricts the generalizability of our results to other populations. It is essential to consider this limitation when extrapolating our findings. Due to limitations in the database, we may not be able to cover mid-term results. We will conduct further research in our own studies. Given these limitations, well-designed multicenter controlled trials are imperative to validate the current findings.

## Conclusion

The present study establishes a positive association between RDW and in-hospital mortality in patients diagnosed with both CHF and COPD. This study, for the first time, underscores the prognostic significance of RDW in critically ill patients with CHF and COPD, implying its potential role in risk stratification upon admission. Nevertheless, to bolster the validity of these findings, larger prospective investigations with extended follow-up periods are warranted.

## Data Availability

Publicly available datasets were analyzed in this study. This data can be found here: MIMIC.
