# Supplementary material for: Associations between red cell distribution width and in-hospital mortality in congestive heart failure patients with chronic obstructive pulmonary disease: a retrospective cohort study
Source: Front Med (Lausanne). 2025 May 20;12:1448930. doi: 10.3389/fmed.2025.1448930 (PMC12129970; doi:10.3389/fmed.2025.1448930)
Supplement: Supplementary file 1 [file Table_1.docx]

**Table S1** ICD codes or SQL queries for obtaining ICD codes

| Disease | ICD9 | ICD10 |
| --- | --- | --- |
| CHF | 39891, 40201, 40211, 40291, 40401, 40403, 40411, 40413, 40491, 40493, 4254, 4255, 4257, 4258, 4259, 4280, 4281, 42820, 42821, 42822, 42823, 42830, 42831, 42832, 42833, 42840, 42841, 42842, 42843, 4289 | I509, I099, I110, I130, I132, I255, I420, I425, I426, I427, I428, I429, I43, I50, I501, I502, I5020, I5021, I5022, I5023, I503, I5030, I5031, I5032, I5033, I504, I5040, I5041, I5042, I5043, I508, I5081, I50810, I50811, I50812, I50813, I50814, I5082, I5083, I5084, I5089, P290 |

SQL, Structured Query Language; ICD, International Classification of Diseases; CHF, congestive heart failure;
